# Essential Oils and Their Combination with Lactic Acid Bacteria and Bacteriocins to Improve the Safety and Shelf Life of Foods: A Review

**DOI:** 10.3390/foods12173288

**Published:** 2023-09-01

**Authors:** Danka Bukvicki, Margherita D’Alessandro, Samantha Rossi, Lorenzo Siroli, Davide Gottardi, Giacomo Braschi, Francesca Patrignani, Rosalba Lanciotti

**Affiliations:** 1 Faculty of Biology, Institute of Botany and Botanical Garden ‘Jevremovac’, University of Belgrade, Takovska 43, 11000 Belgrade, Serbia; dankabukvicki@gmail.com; 2 Department of Agricultural and Food Sciences, University of Bologna, 40127 Bologna, Italy; margheri.dalessandr3@unibo.it (M.D.); samantha.rossi2@unibo.it (S.R.); davide.gottardi2@unibo.it (D.G.); giacomo.braschi2@unibo.it (G.B.); francesca.patrignani@unibo.it (F.P.); rosalba.lanciotti@unibo.it (R.L.); 3 Interdepartmental Centre for Industrial Agri-Food Research, University of Bologna, 47521 Cesena, Italy

**Keywords:** natural antimicrobials, food preservatives, biocontrol agents, food safety

## Abstract

The use of plant extracts (e.g., essential oils and their active compounds) represents an interesting alternative to chemical additives and preservatives applied to delay the alteration and oxidation of foods during their storage. Essential oils (EO) are nowadays considered valuable sources of food preservatives as they provide a healthier alternative to synthetic chemicals while serving the same purpose without affecting food quality parameters. The natural antimicrobial molecules found in medicinal plants represent a possible solution against drug-resistant bacteria, which represent a global health problem, especially for foodborne infections. Several solutions related to their application on food have been described, such as incorporation in active packaging or edible film and direct encapsulation. However, the use of bioactive concentrations of plant derivatives may negatively impact the sensorial characteristics of the final product, and to solve this problem, their application has been proposed in combination with other hurdles, including biocontrol agents. Biocontrol agents are microbial cultures capable of producing natural antimicrobials, including bacteriocins, organic acids, volatile organic compounds, and hydrolytic enzymes. The major effect of bacteriocins or bacteriocin-producing LAB (lactic acid bacteria) on food is obtained when their use is combined with other preservation methods. The combined use of EOs and biocontrol agents in fruit and vegetables, meat, and dairy products is becoming more and more important due to growing concerns about potentially dangerous and toxic synthetic additives. The combination of these two hurdles can improve the safety and shelf life (inactivation of spoilage or pathogenic microorganisms) of the final products while maintaining or stabilizing their sensory and nutritional quality. This review critically describes and collects the most updated works regarding the application of EOs in different food sectors and their combination with biocontrol agents and bacteriocins.

## 1. Introduction

Medicinal plants are a valuable source of new antibacterial, antifungal, and antioxidant compounds due to the large biological and structural diversity of their constituents [1,2,3]. Botanical species and their derivatives, including EOs, extracts, and bioactive compounds (BACs), have been discovered to be key contributors to the pharmaceutical, agricultural, and food industries.

Traditionally, medicinal plants are used to treat diseases, but they are also well suited for the food industry as natural antimicrobial preservatives [4,5,6,7]. Chemicals (benzoates, propionates, sorbates, nitrates, nitrites, etc.) commonly used as food additives are reported to be the cause of health problems such as allergies, asthma, liver damage, and cancer [8,9,10,11,12]. These concerns reinforce the interest in using natural antimicrobials in food formulations.

**Figure 1:**
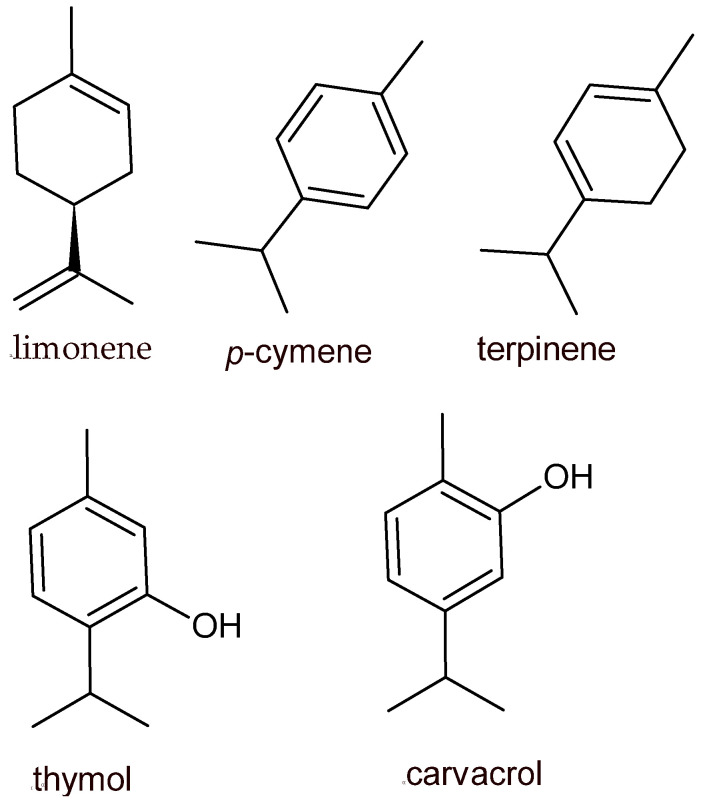
Chemical structures of the most applicable EO components in foods.

Alcohols, ethers or oxides, aldehydes, ketones, esters, amines, amides, phenols, heterocycles, terpenes (an oxygenated derivative of terpenoids), and polyphenols represent the chemical classes to which belong the constituents of EOs and plant extracts. Some terpene compounds identified in EOs are limonene, β-caryophyllene, *β*-pinene, α-pinene, α-terpinene, sabinene, *β*-myrcene, *γ*-terpinene, cinnamyl alcohol, *δ*-3-carene, and *p*-cymene. Limonene is mainly distributed in citrus EOs: orange [13], grapefruit [14], and pomelo [15]. Thymol, carvacrol, and eugenol, deriving from *Thymus* [16], *Origanum* [17], and *Ocimum* [18], represent the main phenolic terpenes associated with EOs obtained from medical and aromatic plants (Figure 1).

A large variety of these compounds are recognized to have strong and effective antimicrobial activity [19,20,21,22]. EOs from the *Lamiaceae* family aromatic plants (Table 1) (i.e., oregano and thyme) and their constituents (like carvacrol and thymol) have been described in the literature as potential preservatives with significant effects on food shelf-life [23,24,25,26,27,28,29]. For instance, clove (*Syzygium aromaticum*), thyme (*Thymus vulgaris*), and rosemary (*Rosmarinus officinalis*) have high antibacterial activity against pathogenic bacteria, including *Staphylococcus aureus*, *Bacillus cereus*, *Escherichia coli*, *Salmonella enteritidis*, and *Pseudomonas aeruginosa*, and can be used safely as food preservatives [30]. Plant-based EOs and extracts’ antibacterial modes of action have been discovered to be dependent on their influence on bacterial cell membranes by lowering cytoplasmic pH and promoting cell membrane hyperpolarization [30].

The lipophilic nature of EOs is directly related to their antibacterial activity. EOs destabilize the membrane potential of bacterial cells by disrupting the permeability of the plasma membrane [31]. Degradation of membrane lipid fractions by the EO component thymol leads to destabilization of membrane permeability [32]. EOs affect quorum sensing signaling and can inhibit cell-to-cell communication, e.g., biofilm formation in bacterial cells [33]. Bouyahya et al. (2019) [34] reported the antimicrobial activity of *Origanum compactum* EO in the context of disrupting cell membrane stability and integrity and increasing membrane permeability, leading to leakage of cellular material (DNA and RNA). Moreover, the effect of EOs on inhibiting biofilm formation in bacterial communities triggers the breakdown of their sensing communication [35]. Few studies report that the mechanisms of action of phenolic compounds in EOs are mainly related to their activity on cell viability and interaction with transcriptional regulators of quorum sensing communication and biofilm formation [36,37]. In addition, EOs can inhibit cell-cell communication and biofilm formation in bacterial cells [33,35]. Martínez et al. (2021) [38] reported the inhibitory effect of *Lippia origanoides* and *Thymus vulgaris* EOs (containing thymol–carvacrol) against the biofilm formation of *S. aureus* and *E. coli*. In addition, EOs cause leakage of cytoplasmic materials and damage to the plasma membrane, leading to the efflux of DNA, RNA, and proteins in bacteria [39]. Han et al. (2020) [40] investigated the antibacterial activity of limonene against *Listeria monocytogenes* and concluded that limonene at a concentration of 20 mL/L can destroy the cell wall and cell membrane, leading to leakage of proteins and nucleic acids. By inhibiting ATPase activity, limonene could inhibit ATP synthesis and respiratory chain complex activity. The effect of EOs during biosynthesis occurs via proton motive force through degradation of the plasma membrane, potential disruption of the electron transport system, proton pump disintegration, and ATP depletion [34].

Several EOs, including those of thyme, marjoram, oregano, basil, ginger, lemongrass, and clove, were shown to be highly effective in inhibiting spoilage bacteria in meat [41], dairy products [42,43,44,45,46], and beverages [47]. It is generally accepted that plants are an important source of antimicrobial metabolites, including flavonoids, phenolic compounds, tannins, terpenoids, saponins, and alkaloids. Other than being endowed with antibacterial and antioxidant properties, BACs may help to enhance the food’s sensory and organoleptic properties and its acceptability, including its shelf life [48].

**Figure 2:**
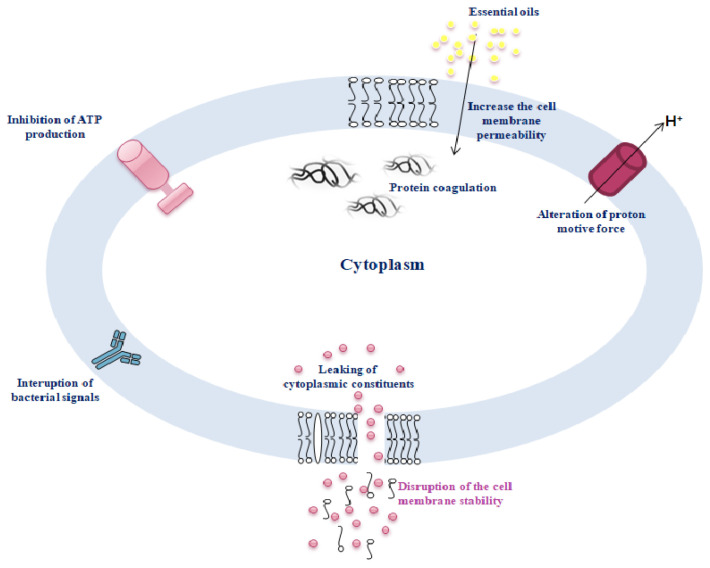
The antimicrobial mechanisms of action of essential oils.

Many EOs and plant extracts have the GRAS (generally recognized as safe) status obtained by the Food and Drug Administration (FDA) and the European Food Safety Authority (EFSA), (Table 1 and Figure 2). Therefore, they can be applied in the food industry, meeting in this way the consumer requirement for natural food preservatives [4].

However, despite EOs positive effect, their practical use as effective antimicrobial agents in the food sector is still a challenge due to their volatile characteristics, hydrophobicity, and low stability. Nowadays, new technologies and delivery strategies such as nanoencapsulation, encapsulation in active packaging, or polymer-based coatings have efficiently addressed these issues and improved the efficacy and gradual release of EOs [49]. Eventually, EO application can be limited by the sensory impact it imparts when applied at the required active concentrations. Therefore, a possible solution to overcome this aspect is their combination with other natural strategies. Among the ones proposed for increasing the safety and shelf-life of fruit, vegetables, meat, and dairy products is the application of safe microorganisms, including lactic acid bacteria (LAB) and yeasts and their metabolites, as biocontrol agents to inhibit the growth of pathogenic and spoiling microorganisms [50]. Due to its GRAS status, LABs are largely the most commonly used biocontrol agents; however, yeasts are mainly used as biocontrol agents to control postharvest diseases. The mechanism of action of LABs is linked to several factors that include competition for space and nutrients, the production of organic acids that can enter microbiological cells, lowering the internal pH, causing enzyme denaturation, disruption of the cell membrane, and the collapse of the proton motive force [51]. In addition, LABs are reported to produce hydrogen peroxide, this compound is a strong oxidant capable of causing oxidative damage to microbial cells, mainly targeting thiol groups within enzymes [51,52]. Diacetyl is also an aromatic molecule largely produced by LABs, able to exert high antimicrobial activity, especially against Gram-negative bacteria, and whose mechanism of action is mainly attributed to protein damage and interference in arginine metabolism [53].

Finally, LABs can produce different classes of antimicrobial peptides called bacteriocins. These molecules are antimicrobial peptides produced by specific bacterial species to protect themselves from other bacteria by inhibiting or killing them without harming themselves. The bacteriocins action mechanism is mainly associated with the permeabilization of the cell membrane, but it is reported that they can also act on protein metabolism, DNA and RNA, and quorum sensing [54,55]. The application of bacteriocins in the food sector has increased in recent years. They have attracted noticeable attention for their potential application as natural and safe food preservatives since they are easily digestible in the gastrointestinal tract. Nisin represents the most known and studied bacteriocin; it is produced by *Lactococcus lactis,* and its use was authorized by the Food Drug and Administration (FDA) in more than 50 countries [56]. Three ways of bacteriocin applications in foods are reported: inoculation of bacteriocin-producing LABs directly in the food products (starter or protective cultures), application as a food additive, and incorporation in food packaging of the purified or partially purified bacteriocin. Several studies have investigated the direct application of LABs as potential biopreservatives due to their ability to produce antimicrobial compounds such as bacteriocins, other bioactive peptides, organic acids, and hydrogen peroxide [57]. The great advantage of this strategy is represented by the lesser legal restriction on the use of LABs cultures in foods compared to the directed use of pure or refined bacteriocins [58,59]. Combining EOs, or their components, with protective microorganisms has been proposed as a promising approach to enhancing the shelf life of ready to eat fruits and vegetables. The combined use of some EOs and LABs or LABs products, such as bacteriocins, can be very effective against food pathogens and may improve the sensory properties. In fact, the combination of EOs with biocontrol agents may reduce the EO concentration necessary to achieve sufficient antimicrobial activity and can have a synergistic effect. For example, Iseppi et al., 2020 [60] tested *T. vulgaris*, *S. officinalis* EOs, and bacLP17 alone and in combination to control the growth of *L. monocytogenes* in seafood and proved that the combined use of EOs and bacteriocins produced synergistic effects and overcame the sensory effects of EOs. In addition, Turgis et al. (2012) [61] tested six EOs and four bacteriocins (nisin, pediocin, and two bacteriocins isolated from *Enterococcus faecium*) against foodborne pathogens. The combination of nisin and *O. vulgare* EO produced a synergistic effect against *L. monocytogenes*, while nisin and *T. vulgaris* EO together produced a synergistic effect against *S. typhimurium*. Two bacteriocins in combination with thyme EO and one in combination with rosemary EO showed additive activity against *L. monocytogenes* and *E. coli* O157:H7 [62]. In the following chapters, an overall description of EOs and protective cultures, applied alone or in combination, will be provided. Moreover, their applications in real food systems will be critically described.

**Table 1:** Biological activities of medicinal plant products and their components in food model systems.

Botanical/Common Name	Plant Product	Bioactivity in Food Model/Product Quality	Reference(s)
*Artemisia dracunculus* L. (Tarragon)	* EO	Antimicrobial in beef burger/flavor enhancer in meat products	[63]
*Allium sativum* (Garlic)	EO	Antibacterial/in poultry meat	[64]
*Allium schoenoprasum* (Chives)	Diallyl sulfides	Inhibits the growth of foodborne pathogens	[65]
*Anethum graveolens* (Dill)	EO	Antimicrobial in dairy products/improves the physico-chemical and sensory characteristics of yogurt	[66]
*Brassica nigra* (Black mustard)	Extracts combined with oregano, *Syzygium,* and cinnamon	Antimicrobial, antioxidant/chicken meat/improve sensory attributes	[67]
*Carum carvi* (Caraway)	* EO	Improves the quality of dry-fermented sausages/reduces the level of sodium nitrite in dry-fermented sausages	[68]
*Citrus aurantifolia* (Lime)	* EO, Limonene, β-Pinene, γ-Terpinene, and Citral	Antimicrobial	[69]
*Crocus sativus* (Saffron)	Stigma powder	Antimicrobial, antioxidant/chicken breast meat/improves physico-chemical characteristics of chicken meat	[70]
*Curcuma longa* (Turmeric)	Rhizome extract/curcumin	Oxidative stability of meat increases the shelf life and quality in meat.	[71,72]
*Cuminum cyminum* (Cumin)	* EO, cuminal	Antibacterial/meet protection/prolongs the shelf life	[73]
*Cymbopogon citratus* (Lemon grass)	* EO combined with ginger EO	Antimicrobial in fresh chicken meat/extends the shelf life of chicken meat for 9 days at a temperature of 2–7 °C	[74]
*Foeniculum vulgare* (Fennel)	* EO	Antioxidant and antimicrobial nanocoating of fennel EO in meat/fish packaging/improves the antioxidant and antimicrobial properties of coatings	[75]
*Hyssopus officinalis* (Hyssop)	* EO combined with coriander EO	Prolongs the shelf life of ground beef/preserve in vacuum-packed meat	[76]
*Kaempferia galanga* (Kencur)	Extract	Antibacterial activity in poultry products/cell membranes damage in food pathogen bacteria	[77]
*Laurus nobilis* (Bay)	* EO/leaf extract	Antibacterial/increase the shelf life of lamb meat/increases the shelf life of lamb meat	[78]
*Lippia graveolens* (Mexican oregano)	EO mixed with basil EO	Microencapsulated EOs increases the shelf life of refrigerated meat products and positive effect on the sensory properties	[79]
*Mentha piperita* (Mint)	* EO	Prolongs the shelf life of beef meat/extends the shelf life	[80,81]
*Melissa officinalis* (Balm)	* EO combined with thyme EO	Antimicrobial/may protect the chicken meat from decomposition during storage and extends the shelf life	[82]
*Ocimum basilicum* (Basil)	* EO with aloe vera	Extend the shelf-life of strawberry fruit and preserve post-harvest quality Combinations of EOs of *O. basilicum*, *Cymbopogon nardus* and *C. flexuosus* increased banana shelf life by up to 21 days/control post-harvest diseases, and extended storage life.	[83,84]
*Murraya koenigii* (Curry leaf)	Leaf powder	Antioxidant/cooked goat meat/inhibitor of oxidation products in raw ground and cooked goat meat	[85]
*Myristica fragrans* (Nutmeg)	* EO	Antibacterial and antioxidant/improves the color stability and sensory properties of beef slices	[86]
*Nigella sativa* (Black cumin)	EO	EO prolongs the shelf life and improves the sensory quality of fresh fish fillets	[87]
*Origanum vulgare* (Oregano)	* EO/carvacrol	Antibacterial/increases the shelf life of pork, antimicrobial oregano oil nanoemulsions in fresh lettuce, antibacterial in vacuum-packed minced beef	[23,88,89]
*Pimpinella anisum* (Anise)	* EO	Antimicrobial/antioxidant/prolongs the shelf life of chicken fillets	[90]
*Rhus coriaria* (Sumac)	Water extract	Antimicrobial effect/extends shelf life of refrigerated raw broiler wings, improving sensory quality, and color	[91]
*Rosmarinus officinalis (Rosemary)*	* EO	Antimicrobial against meat pathogens/extends the shelf life of beef stored at 4 °C for 20 days	[92]
*Salvia officinalis* (Sage)	* EO Ethanolic extracts Hydro-ethanol extract Oil and ethanol extracts	Prolongs the shelf life and compositional quality of fish burgers (4 months at frozen storage) Antioxidant/extends the shelf life of mayonnaise during storage Prolongs the shelf life of trout fillets Antibacterial/prolongs the storage stability of vacuum-packed low pressure chicken meat	[93,94,95,96]
*Satureja montana* (Winter savory)	EO/supercritical extracts	Antioxidants in pre-cooked pork chops/extends the shelf life	[97]
*Thymus vulgaris* (Thyme)	* EO	Antimicrobial/prolongs the shelf life of gilthead seabream extends the shelf life of oranges for fresh use and juice processing	[47,98]
*Thymus capitatus* (Headed Savory)	EO	Antimicrobial/improves the microbial and sensory quality of beef meat	[99]
*Trachyspermum ammi* (Ajwan)	EO	Antibacterial/chitosan-based film with EO improves the safety of chicken fillet stored for 12 days at refrigerated temperature	[100]
*Zingiber officinale* (Ginger)	* EO	Antimicrobial/extends the shelf life of minced meat	[101]

* EOs—Essential Oils considered generally safe by the FDA—GRAS. https://www.biosourcenaturals.com/pure-essential-oils/essential-oils-considered-safe-by-the-fda/ (accessed on 28 July 2023)

## 2. Use of Essential Oils and Biocontrol in Minimally Processed Fruit and Vegetables

Over recent years, the market for minimally processed fruits and vegetables has increased steadily. Their popularity is due to their convenience, reduced waste production, and high nutritional content. In fact, these foods represent a source of valuable compounds such as vitamins, minerals, fibers, and antioxidants and may help prevent chronic diseases. Nevertheless, fruits and vegetables are a suitable matrix for microbial proliferation [102,103]. In fact, these foods can be easily spoiled due to the high nutrient and water content and the numerous processing steps that fresh produce undergoes (i.e., peeling, cutting, or slicing), which can promote microbial proliferation by releasing nutrients and transporting the microbial population present on the surfaces of vegetables and fruits into the cut ones [104,105]. In addition, several outbreaks of foodborne illness in the last twenty years were attributed by the FDA and EFSA to the consumption of fruits, vegetables, and fresh juices. In particular, fruits and vegetables have been associated with outbreaks of listeriosis, salmonellosis, and Shiga toxin-producing *E. coli* at EU and international levels (EFSA and ECDC). In addition, literature data indicated the potential presence of pathogens, including *Salmonella* spp., *Listeria monocytogenes*, *Aeromonas hydrophila*, *Yersinia enterocolitica*, *Staphylococcus aureus*, and *E. coli*, on fresh fruit and related minimally processed products [103].

Currently, the use of chemical sanitizers in the washing step, as well as the maintenance of refrigerated temperatures during processing and storage, are the only stages capable of reducing and controlling the microbial population in minimally processed vegetables [106]. However, the use of chemical sanitizers, especially chlorine-based ones, is not always effective, can result in the production of toxic molecules, and is not well accepted by consumers [88]. Because of these drawbacks, researchers are looking to alternative sanitizers (i.e., ozone, hydrogen peroxide, and peroxyacetic acid), physical treatments (UV light, ultrasound, and gamma rays), and natural food additives. As a result of their antibacterial and antioxidant properties as well as their GRAS classification, EOs have the potential to extend the shelf life of several foods, including fresh-cut fruits and vegetables. EOs can be used in various stages of food and vegetable processing, including the washing step, directly on the product, or in the packaging. In fact, the incorporation of EOs and their components in active food packaging is of high relevance. Active packaging is a solid matrix from which EOs are gradually released during food storage. Generally, various methods, including direct incorporation, coating, and surface modification, are applied to incorporate EOs into active packaging [107]. Antimicrobial active packaging is designed to enhance product safety and shelf life by inhibiting or reducing microbial growth in packaged foods [92,108,109]. In this context, it is fundamental to use suitable solvents or polymeric carriers, as EOs are always used in diluted form, which should be food-grade and not interfere with the antimicrobial and antioxidant activities of EO constituents [110].

Several studies have demonstrated the potential of natural antimicrobials, including EOs, to improve the quality and safety properties of processed fruits and juices (Table 2). Among the EOs, those derived from citrus fruit peels are the most interesting. Citral (3,7-dimethyl-2-7-octadienal) contains two isomers, geranial and neral, and is often applied as a flavoring agent in citrus drinks. It is characterized by strong antimicrobial activity. Low concentrations of citral and citron EO were shown to be effective in increasing the shelf life of fruit-based salads in syrup [111] and improving the stability of fruit-based beverages. Hexanal and 2-(E)-hexenal, which are volatile compounds produced by various fruits and vegetables, are also interesting compounds that have already been tested as antimicrobials in minimally processed fruits and vegetables [112]. These molecules are characterized by strong antimicrobial activity against spoiling yeasts, and the addition of these molecules in the storage atmosphere of fresh-cut apples resulted in an extended shelf life [92]. In addition, these molecules also have a positive impact on sensorial properties and exert antioxidant activity that prevents browning of the packaged products. These compounds showed remarkable antibacterial activity against pathogens such as *Salmonella* spp., *E. coli*, and *Pseudomonas aeruginosa* [50].

Other researchers found that dipping sliced apples in a solution containing dissolved hexanal (250 ppm) or the combinations hexanal/2-(E)-hexenal and citral/2-(E)-hexenal (125 ppm of each compound) increased quality parameters and extended the shelf life by reducing the proliferation of naturally occurring yeast [102]. In addition, sliced apples treated with hexanal/2-(E)-hexenal, and citral better retained both color and textural properties. The combination with an active, modified atmosphere (7% O_2_ and 0% CO_2_) enhanced the safety and shelf life of minimally processed apples washed with a solution containing a mixture of hexanal/2-(E)-hexenal. Other researchers discovered that active packaging containing thymol and eugenol inhibited the growth of mesophilic bacteria and yeasts and consequently extended the shelf life of table grapes stored in a modified atmosphere (MAP) compared to the control group [108]. Moreover, when compared to a control, a package containing eugenol, thymol, menthol, or eucalyptol was able to reduce mold, yeast, and total aerobic mesophilic bacteria cell loads [113]. An edible coating based on apple puree alginate enriched with lemongrass, oregano, and vanillin reduced the growth kinetics of psychrophilic bacteria, yeasts, and molds on freshly cut ‘Fuji’ apples [105]. Moreover, the same active coating containing lemongrass (1.0 and 1.5% *w*/*w*) and oregano oil (0.5% *w*/*w*) showed the highest antimicrobial activity on *Listeria innocua* (4.0 log reduction) [105]. Abadias et al. (2011) [104] investigated alternative products to reduce the unwanted chlorine by-products in the fresh-cut industry. When used at 5–15 mM in the dipping solution, cinnamic acid and carvacrol were highly efficient in reducing microflora on fresh-cut apples, kiwifruit, and fresh-cut melon [114] (Table 2).

The use of EOs has great potential, even in minimally processed vegetables. In fact, herbal EOs and their constituents were studied as alternative natural disinfectants for reducing the presence of spoiling and pathogenic bacteria. Due to the strong antimicrobial activity in vitro of oregano (*Origanum vulgare*) and thyme (*Thymus vulgaris*) EOs and their main constituents (carvacrol and thymol), these compounds are the most suitable to be used in minimally processed vegetables [31,54,112,119,120].

For example, the inclusion of oregano and thyme EOs at a concentration of 250 ppm in the washing solution of minimally processed lamb’s lettuce resulted in a product shelf life comparable to the one obtained by using chlorine (120 ppm) in the washing step [119]. In fact, the use of EOs resulted in a preliminary decrease in the microbial population. In addition, the color and turgidity of the lettuce were not affected throughout the storage period while the sensorial properties were not negatively affected [119].

When used in the washing solution, oregano oil (*Origanum onites*) was able to inactivate *Salmonella typhimurium* inoculated on iceberg lettuce. The effectiveness of oregano EO treatment (75 ppm) was comparable to that of chlorine treatment at 50 ppm [122]. A new antimicrobial active packaging made of polypropylene (PP)/ethylene-vinyl alcohol copolymer (EVOH) film and activated with oregano EO or citral, combined with a modified atmosphere, was successfully tested to improve the shelf life and safety of packaged salads [115]. Indeed, the addition of oregano or citral reduced the cell load of *Enterobacteriaceae*, yeasts, and molds by about 2 log cycles. This active packaging also decreased the cell load of aerobic bacteria, lactic acid bacteria, and psychrotrophic bacteria. Citral-based films, on the other hand, showed better antimicrobial activity and sensory properties compared to oregano-based films.

When oregano EO was applied to ready to eat lettuce and carrots, the initial decontamination effect was comparable to that of chlorine [127]. Furthermore, oregano EO did not negatively affect the color, texture, or water activity of the samples. However, sensory acceptance of the product treated with oregano EO was only observed for carrots. Volatile antimicrobial compounds such as borneol, carvacrol, cinnamaldehyde, eugenol, menthol, thymol, and vanillin were able to inhibit the growth of *Bacillus cereus* inoculated in carrot juice. Direct application of EOs in the food system has some drawbacks due to their strong odor, chemical reactivity, hydrophobicity, low solubility, and potential negative interaction with the food matrix, leading to alteration of organoleptic properties. Several technological approaches have been tested for EO delivery in food. EO encapsulation is a novel and advanced delivery system. It provides enhanced antimicrobial efficacy as well as control over the release of EO flavors into the food system [128]. Numerous biopolymeric matrices, including chitosan, alginate, starch, cellulose, and dextran, are characterized by excellent biodegradability, biocompatibility, and non-toxicity; for these reasons, they were proposed as carriers for the encapsulation of EOs [49]. Consequently, the encapsulation of EOs represents a non-toxic and environmentally friendly technology.

Several studies reported that various lactic acid bacteria strains, including *Lacticaseibacillus casei*, *Lactiplantibacillus plantarum*, *Leuconostoc* spp., *Pediococcus parvulus*, and *Lactococcus lactis*, are able to enhance the shelf life and safety of minimally processed lettuce, carrots, and apples [129]. LAB isolated from the same food matrix in which they are applied have the best chance of being used as biocontrol agents [120]. Furthermore, combining bioprotective cultures with additional hurdles such as EOs can have an additive or synergistic effect. Siroli et al., 2015, [120] isolated and characterized LAB from minimally processed vegetables and selected *Lactiplantibacillus plantarum* V7B3 and *Lacticaseibacillus casei* V4B4 as potential biocontrol agents in combination with thyme EO in lamb’s lettuce, demonstrating that the addition of *Lactiplantibacillus plantarum* V7B3 in the washing step increased product shelf life and safety compared to the control. Furthermore, the nisin producer strain *Lactococcus lactis* CBM21, whether combined or not with thyme EO and supplemented to the washing solution of minimally processed lamb’s lettuce, reduced the cell load of *L. monocytogenes* and *E. coli*, as well as the total aerobic bacteria, without affecting the color parameters or sensory attributes [118]. The effect of biocontrol agents has been widely assessed in minimally processed fruit, also in combination with modified atmospheres, natural antimicrobials [119], gamma radiation [130], reducing agents [131], and heat treatments [132].

The use of LAB as bioprotective cultures in minimally processed fruits is more limited than in vegetables due to the higher sugar content combined with the low pH, which favors yeast growth with respect to bacteria. Indeed, *L. mesenteroides* and *L. citreum* have been tested as potential biocontrol agents on ready to eat Golden Delicious apples [133]. These strains strongly reduced the growth rate of *L. monocytogenes*. Other authors have selected LAB strains from ready to eat apples and tested them as bioprotective cultures in ready to eat apples (Golden Delicious) packaged in a modified atmosphere and combined with the addition of 2-(E)-hexenal/hexanal and 2-(E)-hexenal/citral [120]. The strain *Lactiplantibacillus plantarum* CIT3, added to the apple dipping solution, was able to extend the shelf life by reducing the yeast growth. The combination of *Lactiplantibacillus plantarum* with 2-(E)-hexenal/citral increased the antimicrobial activity and reduced the detrimental effect on color parameters due to the antioxidant activity of the natural antimicrobials. Moreover, [119,134] tested a *L. lactis* strain as a potential biocontrol agent for sliced apples in combination with the mixtures 2-(E)-hexenal/hexanal and/or 2-(E)-hexenal/citral. This approach improved the product’s safety as well as its shelf life. In fact, *L. monocytogenes* was significantly inhibited, especially when the biocontrol agent was combined with natural antimicrobials.

Although interest in the use of bioprotective cultures in ready to eat fruits and vegetables has increased recently, a critical review of the literature shows that the efficacy of bioprotective cultures, regardless of the microorganism used, is strongly influenced by the inoculum level, the naturally occurring microflora, the product’s physical-chemical properties and composition, and the conditions of storage. Because of the numerous variables involved, standardizing bio-protective methods and, as a result, their industrial scaling up is difficult.

## 3. Application of Essential Oils as Meat and Dairy Preservatives

### 3.1. Antimicrobials as Meat Preservatives

Fresh meat and fresh meat produce are extremely perishable and prone to oxidative and microbial spoilage. These products are easily subject to oxidation, which reduces nutritional value, affecting lipids, proteins, myoglobin (pigments), texture, and flavor [135]. Plant extracts are a natural source of compounds with antioxidants and antimicrobial activity that can be used as an alternative to synthetic ones. EOs and their constituents have been successfully used in meat and meat products to prevent oxidation, degradation, and microbial proliferation [136,137,138]. Promising results can be achieved with EOs from the *Lamiaceae* family, including oregano, thyme, sage, and rosemary, which are commonly used as flavoring agents and prevent oxidative degradation. The antioxidant activity of EOs is well documented and can be attributed to phenolic compounds including eugenol, carvacrol, and thymol, which act as hydrogen donors and scavengers of free radicals [139,140].

Fasseas and colleagues (2007) [141] measured the antioxidant activity of pork and beef meats (raw and cooked) treated with sage and oregano EOs over a 12 day storage period at 4 °C. Compared to controls without added EOs, the EO-treated meat (raw and cooked) had higher TBA (thiobarbituric acid) scores and 2,2-diphenyl-1-picrylhydrazyl (DPPH) radical scavenging activity. However, oregano EO showed higher antioxidant activity than sage EOs (*p* < 0.001) both in raw and cooked meat samples [141].

Estévez et al. (2007) [142] reported that 0.1% of sage and rosemary EOs improved the lipids stability to oxidation in refrigerated stored liver pates (90 days). The effects were primarily attributed to reduced polyunsaturated fatty acid degradation, which prevented the synthesis of lipid-derived volatiles and residual components such as malonaldehyde. However, the application of EOs as antioxidants in meat and meat derivates requires deeper research into their properties and antioxidant mechanisms of action [139]. Some EOs can promote meat oxidation, depending on their concentration. The oxidation of the phenolic compounds of EOs to phenoxyl radicals could lead to new degradative reactions [143].

Meat contaminated with spoilage microorganisms shows color changes, the development of uncharacteristic and undesirable odors and tastes, and the formation of superficial slime [144,145]. Bacteria belonging to the genera *Pseudomonas*, *Acinetobacter*, *Staphylococcus*, *Brochothrix*, *Moraxella*, *Micrococcus*, and *Flavobacterium*, as well as LAB and genera belonging to the *Enterobacteriaceae* family, are reported to be involved in the spoilage of meat and meat derivatives [145,146]. In addition to spoilage microorganisms, poultry, pork, beef, sheep, and other animal meat consumed by humans can also be reservoirs or carriers of foodborne pathogens. In fact, it has been reported that the ingestion of contaminated meat can transmit enteropathogenic bacteria, including *Escherichia coli*, *Salmonella* spp., *Yersinia enterocolitica*, *Shigella* spp., *Campylobacter*, *Clostridium botulinum*, *Listeria monocytogenes*, *Staphylococcus aureus*, and *Vibrio* spp. [147,148,149].

The antimicrobial properties of EOs are generally dependent on their chemical composition. As mentioned earlier, in addition to phenolic compounds, EOs are a complex mixture of terpenes (mono- and sesqui-), terpenoids, alcohols, ketones, aldehydes, esters, and other aromatic and aliphatic compound classes [150]. A literature review indicated that EOs can be used as antimicrobials against both meat spoilage agents and pathogens. Some examples are given in this section.

Cell loads of foodborne pathogen *L. monocytogenes* deliberately inoculated on ham (3 log CFU/g) were decreased by 10 and 19% after supplementation of *Cinnamon cassia* and oregano EOs, respectively [151]. *Cinnamon cassia* EO was also effective in promoting the shelf life of ground lamb meat throughout refrigerated storage at 4 °C. Compared to control samples, supplementation of *Cinnamon cassia* EO (0.5%) reduced from the 4th to the 16th day the lactic acid bacteria spoilage population up to 1.9 log CFU/g and *Enterobacteriaceae* up to 1.1 CFU/g [152]. A reduced microbial spoilage population was also observed in minced meat with rosemary, thyme, and oregano EOs at concentrations between 1 and 1.5% (*v*/*w*). Compared to the control group, treated samples showed reduced loads of LAB, molds, and yeast [153]. Thyme EOs tested at two different concentrations (0.02 and 0.05 *w*/*w*) inhibited the growth of coliform in chicken hamburgers [154].

The use of EOs as antioxidants and preservatives for meat is dependent on the bioactivity and stability of their components. Most EO constituents lose physical stability when they interact with meat constituents [155], probably due to the binding ability of fats and proteins in meat to volatiles in EOs [155]. When encapsulated EOs are added to edible coatings, plastic films, or meat during marinating, their stability increases significantly. The possibility of using active packaging supplemented with natural antioxidants and antimicrobials has recently been investigated [58,156]. The use of milk protein-based film added with 1% (*w*/*v*) of oregano EO led to a shelf life extension and the inhibition of foodborne pathogens (*E. coli* O157:H7 and *Pseudomonas* spp.) in beef slices [157]. Oregano oil also showed a good efficacy, when incorporated into a whey protein-based coating, to extend the shelf life and microbial stability of Portuguese sausages without affecting the sensorial properties of the product. In addition, a reduction in oxidation of the lipid fraction was also observed [158] (Table 3).

The stability, antioxidant, and antimicrobial properties of rosemary EO can be enforced by encapsulation with nanogel. Compared to samples treated with free rosemary EO, rosemary EO encapsulated in chitosan-benzoic acid-based nanogels showed greater efficacy in reducing the cell load of *Salmonella typhimurium* inoculated on beef chops during storage at 4 °C [128]. Chitosan-based coatings also improved the performance of oregano EO in reducing lipid oxidation in dry fermented sausages in comparison to control samples after 7 months of storage [159].

Another strategy to improve EO stability is to convey EOs during the meat marinating process. Siroli et al. (2020) [160] used an oil/beer/lemon marinade solution containing oregano, rosemary, and juniper EOs to improve the shelf life and food safety of pork loin slices. The marinade supplemented with EOs reduced the growth of *Salmonella enteritidis*, *Listeria monocytogenes*, and *Staphylococcus aureus*. A water solution of sodium lactate/lactic acid buffer (2%) and NaCl (10%) enriched with a combination of EOs obtained by cinnamon (*Cinnamomum zeylanicum*), thyme (*Thymus zygis*), and oregano (*Oreganum compactum)* was able to increase the shelf life of pork fillet, whereas this approach was not effective for chicken breast fillet [161].

LABs can be added for two different purposes: as starting materials for producing fermented meat products or as a bioprotective culture used only to compete with naturally occurring microflora and produce antimicrobial peptides [59]. Many studies have investigated the application of strains belonging to *Pediococcus acidilactici*, *Latilactobacillus curvatus*, and *Latilactobacillus sakei* in various cured meats from the Mediterranean region to counteract the growth of pathogens including *Salmonella* spp., *E. coli*, *Listeria* spp., and *S. aureus* [162]. Bacteriogenic cultures of *L. carnosum*, *Lactococcus Lactis*, *L. sakei*, and *L. curvatus* sprayed on the surfaces of vacuum-packed chicken or beef, ham, bacon, and fermented sausages have been shown to inhibit spoiling microorganisms (*Enterobacteriaceae* and *B. thermosphacta*) and also inhibit the growth of *Listeria* [59,163,164]. Some commercial products are also currently available on the market (Bactoferm™ F-LC, commercialized by Chr. Hansen, and ALCMix1, commercialized by Danisco DuPont).

Although the use of bacteriocinogenic LAB is an appropriate approach for meat products, not all meat products can provide suitable environmental conditions to sustain the culture’s growth and bacteriocin production.

Direct addition of bacteriocins to meat products may result in some loss of their activity caused by various factors, such as dilution phenomena leading to depletion of antimicrobial peptides [165] or inactivation due to interaction with lipids and enzymes present in meat [164,166].

Since the meat is generally contaminated by microorganisms on its surface, the inclusion of bacteriocins on active packaging represents an efficient solution.

The constant release of antimicrobial peptides from the packaging to the product surface could help to maintain the bacteriocin concentration at the optimal level to carry out their bioprotective effect [165]. In various studies, films containing the bacteriocin nisin have been successfully used to preserve meat products [59]. Pullulan film containing Nisin Z, which was used for the packaging of refrigerated vacuum-packed raw beef and deli ham, strongly inhibited various foodborne pathogens, including *Salmonella* spp., *S. aureus*, *L. monocytogenes*, and *E. coli* [167]. Furthermore, the efficacy of this nisin/pullulan film was improved by the addition of lauric alginate [167]. Reduced growth of *S. aureus* on chilled sliced beef was observed when packaged with an alginate-based palmitoylated film supplemented with nisin [168]. Nisin-internalized cellulose envelopes demonstrated anti-*Listeria* properties on chilled vacuum-packed frankfurters (sausages) [169,170] and vacuum-packed hot dogs wrapped with a plastic film enriched with nisin. A combination of nisin and Nisaplin^®^ (a commercial product from DuPont) adsorbed in cellulose-based packaging paper was shown to inhibit *S. aureus*, *L. innocua*, and LAB on cooked ham [171].

The use of a combination of biocontrol agent/bacteriocins and EOs, or plant actives, in a real food system is not common. Ghalfi et al., 2007 [172] reported that bacteriocin from *Latilactobacillus curvatus* combined with oregano EO was able to limit the proliferation of *Listeria monocytogenes* in refrigerated (4 °C) pork meat for up to 6 weeks, while the single treatment maintained the cell load of the pathogen under the detection limit only for 3–4 weeks. Other works performed in the real food system concern mainly fish. Abdollahzadeh et al. (2014) [173] reported that the combination of thyme EO (at 0.8% or 1.2%) with nisin (at 500 or 1000 IU/g) decreased the cell load of *L. monocytogenes* below 2.0 log CFU/g in minced fish after two days of storage at 4 °C. No increases in the pathogen cell load were observed during the 12 days of storage considered. Instead, Iseppi et al. (2023) [174] showed that the addition of bacteriocin bacLP17 in edible coating reduced the MIC values of *Salvia officinalis*, *Citrus limon*, *Mentha piperita*, or *Thymus vulgaris* EOs against *L. monocytogenes* when tested on artificially contaminated shrimps.

**Table 3:** Short overview of applications of EOs and combinations of EOs with bacteriocins/nisins in meat or meat products.

Microbial Target	Meat/Meat Products	EO or Combinations	Concentration Used/Product Benefits	Reference(s)
*Listeria monocytogenes*	Ham	Oregano and *Cinnamon cassia* EOs	500 ppm/inhibit the growth of food pathogens with no effect on sensory attributes	[151]
Enterobacteriaceae	Lamb meat	*Cinnamon cassia* EO	(0.5%) (*w*/*w*)/reducing bacteria growth but worse color stability	[152]
*S. enteritidis*, *Listeria monocytogenes*, *St. auresu*	Marinated pork loin	Oregano, rosemary, and juniper EO	(0.02–0.03%) (*w*/*w*)/extends shelf life, Improve sensory characteristics	[160]
*Salmonella typhimurium*	Pork meat	*Micromeria dalmatica* EO	0.15 mg/mL/reduces bacterial growth	[175]
*E. coli*, *Salmonella* sp.	Chicken breast meat	Thyme and balm EO	(0.5%) (*w*/*w*)/extends the shelf life	[82]
*E. coli*	Chicken hamburgers	Thyme EO	0.02 and 0.05 *w*/*w*/provides oxidative and microbial stability	[154]
*Salmonella*, *Listeria* and *E. coli*	Sausages	*Oreganum virens* EO	EO incorporated in active film/extends the shelf life and sensory properties	[158]
psychrotrophics, *Brochothrix thermosphacta*, *Pseudomonas*	Beef meat	Rosemary EO	4% (*w*/*w*)/extends the shelf-life of refrigerated beef meat	[92]
*L. monocytogenes*	Pork meat	Oregano EO + bacteriocin	50 µL/100 g/synergistic effect of EO and bacteriocins prolongs the shelf life for two weeks while under storage at 4 °C	[172]
*L. monocytogenes*	Minced fish	Thyme EO + Nisin	EO 0.4% nisin (1000 IU/g)/control bacteria growth and improve sensory properties	[173]
*L. monocytogenes*	Shrimps	*Salvia*, *Citrus*, *Mentha*, *Thymus* EO+ bacLP17	EOs 8-128 µL/mL; bacLP17 16 µL/mL /improve organoleptic properties and reduce growth of *L. monocytogenes*	[174]
*Pseudomonas* spp., LAB and *B. thermosphacta*	Marinated beef	Thymol and carvacrol	0.4% and 0.8% (*w*/*w*)/extends the shelf life	[176]
*Salmonella typhimurium*, *Listeria monocytogenes*, and *Escherichia coli* O157:H7	Beef slices	Thymol	(0.5%) (*w*/*w*)/inhibited growth aerobic bacteria inactivate coliform bacteria	[177]
*Salmonella enteritidis*, *Campylobacter* and *E. coli*	Breast fillets and wings	Marinade with thyme and orange EOs	(0.5%) (*w*/*w*)/inhibit microbes	[178]

### 3.2. Essential Oils as Dairy Preservatives

Plants and spices have been used in cheese production since ancient times, often linked to local traditions. Traditionally, spices and herbs or their extracts were rubbed directly on the cheese. In fact, herbs and spices can be applied as antioxidant, antimicrobial, flavoring, enriching, and functionalizing ingredients, which may improve the appearance and appeal of the product [179].

Although many dairy products undergo heat treatment during their manufacture, they are perishable and can be easily contaminated by microorganisms, resulting in food spoilage, consumer health risks, and a shortened product shelf life. Furthermore, as they are rich in lipids, oxidative processes can lead to a loss of flavor, nutrients, and color, the development of off-flavors, and the accumulation of compounds that may be of concern to human health [180]. For that reason, there is growing interest in the application of plants, extracts, and EOs as natural preservatives in the dairy sector as an alternative to synthetic preservatives [181]. The antimicrobial activity of natural products is commonly assessed against the main pathogens and spoilage bacteria commonly found in the dairy sector, such as *L. monocytogenes*, *S. aureus*, *E. coli*, *Salmonella* spp., and *Pseudomonas* spp., as well as yeasts and molds, such as *Penicillium* and *Aspergillus* [179]. For example, black cumin seed oil supplemented during the manufacturing of a soft cheese inhibited several pathogenic microorganisms generally associated with cheese [182]. The addition of green pepper and cayenne to traditional Egyptian Kareish cheese allowed for a decrease in *S. aureus* cell load [183]. Moreover, the addition in processed cheese of extracts from cinnamon, garlic, lemongrass, cress, rosemary, sage, and oregano resulted in the inhibition of *L. monocytogenes* in processed cheese [184]. Kholy et al., 2017 [185] showed that cumin, rosemary, and thyme EOs were effective antimicrobials, preventing the growth of *S. aureus*, *E. coli*, *Bacillus subtilis*, *B. cereus*, *Salmonella typhi*, and *Aspergillus niger* in ultra-filtrated soft cheese. Clove and cinnamon EOs at 1% were the most effective against *L. monocytogenes* in low-fat cheese within 3 days (≤1 log CFU/mL), while a similar achievement was obtained in full-fat cheese only with clove EO. The same EO was also effective in reducing *S. enteritidis* population mainly in full-fat rather than low-fat cheeses [186]. Bukvicki et al., 2018 [42] showed that the addition of 25 µL of *Thymus algeriensis* EO reduced the incidence of contamination caused by the food pathogenic mold *Penicillium aurantiogriseum* in soft cheese throughout 30 days of refrigerated storage (4 °C). Caleja et al., 2015 [187] increased the antioxidant activity of cheese during 14 days of storage by using *Foeniculum vulgare* decoction. The elevated content of phenolic compounds, but also carotenoids, phenolic diterpenes, flavonoids and anthocyanidins, seems to be the major reason for the strong antioxidant activity of plant-derived compounds [179]. Natural compounds can play a crucial role in increasing food shelf life, but they can also exert a positive effect on consumer health. For instance, Ref. [188] showed that the functional features of cheese can be enhanced after the addition of tomato powder at different concentrations. Fortified cheeses had a higher lycopene content, even after two months of storage. Furthermore, the addition of *Satureja hortenis* to cheese can not only inhibit microbial growth but also stimulate the intake of essential elements (i.e., Fe), which cheese lacks [189].

Natural products have also been proven in additional types of dairy products, including milk. Jemaa et al., 2017 [190] showed that EO of *Thymus capitatus* ameliorated pasteurization effectiveness in maintaining raw milk quality. Incorporation of EOs from basil, peppermint, and zataria in the formulation of probiotic yoghurt increased the inhibition of *E. coli* and *L. monocytogenes* compared to yoghurt without EOs. However, only the EOs of basil and peppermint showed good antioxidant and antiradical activity along with good sensory acceptability [191]. The application of *Echinophora platyloba* EO and lycopene was effective as natural preservatives for dairy products with high fat content, such as butter and cream. Pasteurized cream treated with a mixture of EO and lycopene (0.5% and 50 ppm, respectively) showed improved shelf life (both from a microbiological and chemical point of view) compared to control samples. Sensory evaluation results showed that all the samples have satisfactory overall acceptability, although the greatest sensorial features were detected in creams prepared with a combination of low concentrations of *Echinophora* EO and lycopene (0.1% and 20 ppm, respectively) [192] (Table 4). Other authors extended the shelf life of butter using thyme and cumin EOs [193], while Ozkan et al. (2007) [194] explored the potential of *Satureja cilicica* EO as a natural flavoring and antioxidant in the same dairy product.

Although many studies highlight the beneficial properties of herbal preservatives, other papers report that dairy fats, carbohydrates, or proteins may interact with them and reduce their functional properties. Consequently, higher amounts of EOs are necessary to attain the desired effect, with subsequent drawbacks related to sensorial impacts. To overcome this aspect, different approaches have been investigated. For instance, the use of mixtures of EOs may produce a synergistic effect, determining in turn the requirement for a lower amount of the individual natural compounds.

Nisin is permitted as a food additive for processed cheese by the FAO/WHO Codex Committee at a concentration of 12.5 mg/kg product, while the US FDA is permitted to use up to 250 mg/kg [203]. It is generally known that the effectiveness of nisin is dependent on the dairy products considered. In particular, neutral-pH dairy products made from whole milk are not suitable for nisin use [204]. Nisin, one of the few bacteriocins permitted as a preservative in dairy products, was effective in controlling various pathogens such as *L. monocytogenes* and *S. aureus* in real food matrices (Table 5).

As reported by Arqués et al., 2011, nisin reduced *L. monocytogenes* and *S. aureus* in refrigerated stored milk [205]. Other studies showed the inhibition of *L. monocytogenes* in cheddar, cottage cheese, and ricotta after nisin addition [206]. In addition, its potential against *L. monocytogenes* has also been demonstrated in pasteurized dairy products, including clotted cream, flavored milk, chilled desserts, and evaporated milk. To exploit the synergistic effect of natural compounds (Table 5), Chen et al., 2017 [207] used a ternary combination of nisin (500 IU/mL), thymol (2 mg/mL), and lactobionic acid (10 mg/mL) to reduce *L. monocytogenes* in whole milk.

As already reported for natural plant-derived substances, the activity of bacteriocins can also be negatively affected by interaction with the food matrix. Various approaches have been developed to overcome these shortcomings. Nano- or micro-encapsulation of natural products is a promising approach that guarantees their long-term activity. For instance, rosemary EO was microencapsulated with inulin and whey protein isolate and then incorporated into the Minas Frescal cheese. Alternatively, natural molecules could be incorporated into edible films and coatings. This approach allowed a gradual release over time and long-term antimicrobial activity. For instance, Balaguer et al., 2013 [109] have shown that a film coating incorporated with cinnamon EOs having 5% cinnamaldehyde inhibited the growth of *Apergillus niger* and *Penicillium expansum* on spreadable cheese. So far, a limited number of studies have studied the efficacy of coatings or films added with bacteriocins or bacteriocin-producing LAB in dairy products. However, literature data indicate a reduction in the growth of pathogens when foods were packed with coatings and films enriched with LAB bioactive metabolites [208,209,210,211,212] or containing directly viable LAB cells [213,214].

**Table 5:** Applications of nisin in combination with other natural compounds for the preservation of dairy products.

			Combined Antimicrobial				
Dairy Food Application	Target Microorganisms	Nisin Concentration	Antimicrobial Type	Antimicrobial Concentration	Other Treatment	Activity Reported	Reference
Unpasteurized cow milk	*Escherichia coli*, *S. aureus*	0.008 mg/mL	Magnesium oxide nanoparticles	2 mg/mL without heat	NA *	MgO NP in combination with nisin lead to damage to the cell membrane, causing the pathogen’s death	[215]
				0.5 mg/mL	Heat (60 °C)		
Pasteurized milk	*S. aureus*, *L. monocytogenes*	16 µg/mL	Perilla oil	1 mg/mL	NA *	Synergistic effect resulting in higher cell wall damage when nisin and perilla oil were used in combination	[216]
	*S. aureus*	8 mg/mL	Cinnamaldehyde	0.25 mg/mL	NA *	Synergistic effect with increased antimicrobial activity against *S. aureus*	[217]
		0.37 and 0.75 µg/mL	Phage-encoded endolysin LysH5	7.5 and 15 U/mL	NA *	Synergistic effect of the absence of *S. aureus* was reached only with the combination of the two antimicrobials	[218]
		8 µg/mL	p-Anisaldehyde		NA *	Synergistic effect demonstrated	[219]
		1.5 µg/mL	Bacteriophage Φ35 Bacteriophage Φ88	1:1 cocktail of phages Φ35 and Φ88 at 103 pfu/mL	NA *	nisin activity, which can induce surface changes that can impair bacteriophage activity	[220]
UHT whole milk	*S. aureus*	400, 600, 800, and 1200 AU/mL	Bovicin HC5	400, 600, 800, and 1200 AU/mL	NA *	Bovicin and nisin combinations were effective in inhibiting *Listeria* and *S. aureus* at lower concentrations than when used alone	[221]
	*L. monocytogenes*,						
	*Listeria innocua*,						
UHT processed 2% reduced-fat milk and whole milk	*L. monocytogenes*	250 and 500 IU/mL	Lactobionic acid	10 mg/mL	NA *	LBA increased the synergistic effect between nisin and thymol against *L. monocytogenes* but not *E. coli*	[207]
			Thymol	1–2 mg/mL			
UHT skimmed milk with 0.04% fat	*L. monocytogenes*, *S. aureus*, *E. coli* O157:H7, *Salmonella enterica*, *Yersinia enterocolitica*, *Aeromonas hydrophila*, *Campylobacter jejuni*	100 IU/mL	Reuterin	8 AU6/mL	NA *	Nisin and reuterin showed a synergistic effect in milk at refrigerated temperatures causing the complete deactivation of *Listeria* and *S. aureus*	[205]
Cow milk	*Staphylococcus aureus*, *Listeria monocytogenes*	1/4 MIC	Thymol, eugenol, carvacrol, and cinnamaldehyde	1/4 MIC	NA *	Nisin combination with phenolic compounds showed a synergistic effect	[222]
Whole, low, and skimmed milk	*L. monocytogenes*	62.5, 125, 250, and 500 IU/mL	Cone EO of Metasequoia glyptostroboides	1 and 2%	NA *	Synergistic effect of nisin and cone EO against listeria in whole, low, and skimmed milk	[223]
			Leaf EO of Metasequoia glyptostroboides	1, 2, and 5%	NA *	Remarkable synergism of leaf EO and nisin against listeria in whole, low, and skimmed milk	[197]
Whole (3.5%), low (1%), and skimmed (no fat content) milk		62.5, 125, 250, and 500 IU/mL	Garlic shoot juice	2.5 and 5%	NA *	Synergistic anti-listerial activity of nisin and garlic shoot juice	[224]
Chocolate milk		25 µg/mL	Thymol	100 µg/mL	NA *	Enhanced antilisterial activity by the combination of nisin with carvacrol or cinnamaldehyde	[225]
			Carvacrol	304 µg/mL			
			*trans*-cinnamaldehyde	327.6 µg/mL			
Reconstituted powdered infant milk formula	*Cronobacter sakazakii*	60 µM and 250 µg/mL	Carvacrol	300 µg/mL	NA *	Bioengineered nisin variants showed an increased antimicrobial activity compared to nisin A and an enhanced synergistic effect with carvacrol	[226]
			Citric acid	30 mM			
Homogenized UHT skimmed milk	*S. aureus*	1 to 20 IU/mL	Lysozyme	300 to 5000 IU/mL	High-intensity pulsed-electric field: 120 to 1200 µs	Synergistic effect	[227]
			Enterocin AS-48 (AS-48)	28 AU/mL			
Iranian youghurt (Doogh)	*E. coli O157:H7*	250 and 500 IU/mL	*Ziziphora clinopodioides* Essential Oil	5 mg/mL	NA *	Nisin and EO combination reduced *E. coli* population, showing a synergistic effect	[228]

*: NA—not applicable.

## 4. Conclusions

Essential oils and bioactive compounds can be applied as food preservatives due to their antimicrobial and antioxidant activities, which prevent spoiling processes and guarantee food safety. On the other hand, an additional strategy to increase the safety and shelf life of various types of foods, including minimally processed fruits and vegetables, vegetable beverages, meat, and dairy products, can be represented by the use of protective cultures, especially lactic acid bacteria, which are able to produce antimicrobial compounds such as bacteriocins in addition to organic acids and hydrogen peroxide. Recently, innovative new technologies and delivery strategies such as nanoencapsulation or polymer-based coatings have improved the efficacy and allowed the controlled release of natural antimicrobials. In addition, the use of EOs and bioactive plant molecules in combination with bioprotective cultures or bacteriocins can exert an additive or synergistic effect and reduce the applied concentration of EO. The combination of these antimicrobial agents represents an interesting strategy to increase the shelf life and safety of food due to their antimicrobial and antioxidant properties. The synergistic combinations of EOs and LAB metabolites such as bacteriocins or nisins allow the exploration of promising ways to overcome both the narrow range of antimicrobial action and the unpleasant sensory properties of foods. Future research should focus on the efficacy of different EOs in different food and beverage matrices. At the same time, the combined use of antimicrobial natural products could overcome some of the drawbacks associated with their use in non-combined form. The development of novel strategies such as edible coatings is a great benefit to the environment, as such coatings are biocompatible and environmentally friendly.

## Figures and Tables

**Table 2:** An overview of applications of essential oils (EO) or their components in minimally processed fruits and vegetables.

Microbial Target	Food and Beverages	EO or Component	Concentration Used	Reference(s)
Total aerobic bacteria	Lettuce and carrots Four season salad Table grape Avocado Sweet cherries Kiwifruit and melon Honeydew melon	Oregano and thyme EOs Oregano EO and citral in packaging Eugenol thymol Thyme EO in MAP Eugenol, thymol, and menthol Eucalyptol Carvacrol and cinnamic acid Carvacrol and cinnamic acid	Alone 250 mg/L; combination 125 mg/L 7.5% *w*/*w* 75–150 mL in the gas used for MAP 75 mL in the filter 1000 mL in the gas used for MAP 5–15 mM in the dipping solution 1 mM in the dipping solution	[2,108,113,114,115,116,117]
Total aerobic bacteria and inoculated *L. innocua*	Fresh sliced apples	Oregano, lemongrass, and vanillin used encapsulated	0.5–2.0% (*w*/*w*)	[105]
Total aerobic bacteria, *Escherichia coli*, *Listeria monocytogenes*, *Salmonella enteritidis*	Lamb’s lettuce Fresh cut apples in MAP	oregano and thyme EOs Citron EO, hexanal, E(2)hexenal, citral, and carvacrol	250 mg/L Alone 250 mg/L; combination 125 mg/L	[102,118,119,120]
*Salmonella enteritidis*, *Escherichia coli*, *Listeria monocytogenes*	Fruit salads Fresh sliced apples	citral Citron EO Hexanal, hexyl acetate, E(2)hexenal	25–125 ppm 300–600 ppm 50–250 ppm	[111,117]
*Listeria monocytogenes*, *Yersinia enterocolitica and Aeromonas hydrophilla*	Iceberg lettuce	Oregano and rosemary	0.003–80 mL/m	[103]
*Listeria* spp., *E. coli* O157:H7	Fresh cut apples	Vanillin Oregano EO	12 g/L 0.7–2.1% *v*/*w*	[104,121]
*E. coli* O157:H7	Eggplant salad Carrots	Oregano EO Thyme EO	0.7–2.1% *v*/*w* 0.1–10 mL/L	[106,121]
*Salmonella tiphymurium*	Lettuce	Oregano EO	25–75 mg/L	[122]
*Streptococcus thermophilus*,	Pomegranate juice	*Thymbra capitata* EO	0.06 and 0.125% *v*/*v*	[123]
aerobic mesophilic bacteria				
*Escherichia coli* O157:H7	Apple Juice	*Melissa* oil, carvacrol, and oregano oil,	0.067 and 0.67% *v*/*w*	[124]
*Salmonella enterica*		Terpeineol, geraniol, lemon oil, and citral		
*S. enteritidis*, *E. coli*, *L. innocua*	Apple, pear, and melon juice	Palmarosa, clove, and lemongrass	5–10 μL/mL	[105]
*Saccharomyces cerevisiae*	Orange and pomegranate juices	Cinnamon leaf EO	0.02–0.65 mg/mL	[125]
*S. cerevisiae*, *S. pombe*, *Pichia anomala*	Apple juice	Lemon EO	0.25 μL/mL	[126]

**Table 4:** Use of essential oil/components in dairy products with their sources and reported properties.

Plant Name	Essential Oils/	Activity Reported	Dairy Product	References
	Components Used			
*Mentha piperita*	Peppermint oil	Added functional properties without negative effects on rheological and sensorial properties	Ice-cream	[195]
*Citrus limon*, *Citrus reticulata*, *Citrus aurantium*	*α*- and *β*-Pinene, Limonene, *trans*- *β*-Ocimene, Linalool, α-Terpineol	Increased physiochemical, sensorial, and antimicrobial properties		[196]
*Echinophora platyloba*	Trans-b-ocimene, 2-Furanone, Myrcene, Linalool, *Cis*-b-ocimene	Increase antimicrobial properties and stability	Cream	[192]
*Metasequoia glyptostroboides*	*α*-Pinene, Totarol, *α*-Thujene, Bornylene, *β*-Caryophyllene, *δ*-3-Carene, and 2-*β*-Pinene	Antibacterial and anti-listerial effect	Milk/milk samples	[197]
*Cuminum cyminum*	Cumin oil	Reduce cholesterol, LDL, and increases HDL		[198]
*Thymus capitatus*	Thyme oil	Enhancement of oxidative and fermentative activity, increases physico-chemical, microbiological, and sensory properties		[190,199]
*Thymus vulgaris*	Thymol	Antimicrobial properties		[200]
*Pimpinella anisum*	Anise oil	Increased antimicrobial properties	Yogurt	[201]
*Syzygium aromaticum*, *Salvia rosmarinus*, *Cinnamomum verum*	Eugenol, Acetyl-eugenol, Linalool, β-Caryophyllene, Cineole, Camphor, Camphene, Limonene, *α*-Pinene, *β*-Pinene, *α*-Terpineol, Borneol, and Cinnamaldehyde	Increases shelf life and antioxidant properties		[202]
*Zataria multiflora*, *Ocimum basilicum*, *Mentha piperita*	Zataria, Basil, and Peppermint oil	Antimicrobial and antioxidant properties		
*Satureja cilicica*	Thymol, Carvacrol, *p*-Cymene, and *c*-Terpinene	Increases antioxidant properties and aroma	Butter	[194]

## Data Availability

The data used to support the findings of this study can be made available by the corresponding author upon request.
